# Effects of dietary Chinese herbal mixtures on productive performance, egg quality, immune status, caecal and offspring meconial microbiota of Wenchang breeder hens

**DOI:** 10.3389/fvets.2023.1320469

**Published:** 2023-12-14

**Authors:** Mengjie Liu, Jieyi Huang, Ming Ma, Gengxiong Huang, Yingwen Zhang, Yiqing Ding, Qian Qu, Weijie Lv, Shining Guo

**Affiliations:** ^1^College of Veterinary Medicine, South China Agricultural University, Guangzhou, China; ^2^Guangdong Technology Research Center for Traditional Chinese Veterinary Medicine and Natural Medicine, Guangzhou, China; ^3^International Institute of Traditional Chinese Veterinary Medicine, Guangzhou, China

**Keywords:** Chinese herbal mixtures, Wenchang breeder hens, egg quality, immune status, intestinal microbiota

## Abstract

This study aimed to evaluate the effects of Chinese herbal mixtures (CHMs) on productive performance, egg quality, immune status, anti-apoptosis ability, caecal microbiota, and offspring meconial microbiota in hens. A total of 168 thirty-week-old Wenchang breeder hens were randomly divided into two groups, with each group comprising six replicate pens of fourteen hens. The groups were fed a basal diet (CON group) and a basal diet with 1,000 mg/kg CHMs (CHMs group) for 10 weeks. Our results showed that dietary supplementation with CHMs increased the laying rate, average egg weight, hatch of fertile, and offspring chicks’ weight while concurrently reducing the feed conversion ratio (FCR) and embryo mortality (*p* < 0.05). The addition of CHMs resulted in significant improvements in various egg quality parameters, including eggshell strength, albumen height, haugh unit, and the content of docosatetraenoic acid (C20:4n-6) in egg yolk (*p* < 0.05). The supplementation of CHMs had a greater concentration of IgA and IgG while decreasing the content of IL-6 in serum compared with the CON group (*p* < 0.05). Addition of CHMs to the diet increased the expression of Bcl-2 and IL-4 in liver and ovary, decreased the expression of IL-1β, Bax, and Caspase-8 in jejunum and ovary, and decreased the expression of NF-κB in liver, jejunum, and ovary (*p* < 0.05). Moreover, dietary CHMs reduced the abundance of *Desulfovibrio* in caecal microbiota as well as decreased the abundance of *Staphylococcaceae_Staphylococcus* and *Pseudomonadaceae_Pseudomonas* in the offspring meconial microbiota (*p* < 0.05). In conclusion, the CHMs could improve productive parameters by enhancing immune status, anti-apoptosis capacity, and modulating the caecal microbiota of Wenchang breeder hens, as well as maintaining the intestinal health of the offspring chicks.

## 1 Introduction

A number of announcements from many countries stated that the application of antibiotics in livestock feeds was either strictly restricted or completely prohibited. The production and health state of breeder hens are challenged by more stress factors, including impaired gut function (1) and immune stress (2), which contribute to a downward trend in egg production rate and egg quality. Therefore, it is critical to produce safe feed additives to improve animal health protection. Due to their rich ingredients, Chinese herbal medicines are gaining popularity as dietary supplements for animals (3). Chinese herbal remedies are natural ingredients that have been used in livestock production as safe feed additives to preserve animal health and prevent sickness (4, 5).

A previous study found that *Epimedium* is a flavonoid-rich herb with a range of potential biological activities, such as antioxidant and antibacterial qualities (6). Angelica roots are primarily composed of chemical constituents such as ferulic acid, Z-ligustilide, butylidenephthalide, and various polysaccharides (7). Among these compounds, ferulic acid has anti-inflammatory and immunomodulatory effects (8). *Rehmanniae radix preparata* possesses several pharmacological properties, such as antioxidative (9) and those that enhance immunity (10). The primary components of Radix bupleuri extract consist of saikosaponins, flavonoids, polyacetylenes, and lignans (11), as well as having also been utilized as a remedy for immunomodulatory and antioxidative effects in human and animal species (12). The effective active substances in *Pericarpium citri reticulatae* include flavonoids, total phenols, carotenoids, and polysaccharides (13). The major components of *Leonurus japonicus* include alkaloids, diterpenes, and flavones (14). The pharmacological activities of Yimucao, such as lipid-lowering, anti-inflammatory, anti-oxidative, immunomodulatory, and anti-cancer activities (14, 15). *Paeoniflorin*, a major bioactive constituent in *Radix paeoniae* (16). A study conducted on mice using *Paeonia lactiflora* extract revealed its antioxidant, immunological, and anti-inflammatory effects (17). *Eucommia bark* is abundant in bioactive compounds, including lignans, phenolics, and iridoids. It has demonstrated anti-osteoporosis (18), anti-inflammatory, and antioxidant effects (19). *L. barbarum* polysaccharides are vital bioactive constituents found in *L. barbarum*, known for their diverse range of bioactivities, including antioxidant and immunomodulatory effects (20), as well as liver protection (21). The polysaccharide called *codonopsis pilosula* has generated a lot of attention owing to its prebiotic, antioxidant, anti-tumor, immunomodulatory, and anti-fatigue qualities (22). *Astragalus polysaccharide* has been shown to exhibit antioxidant, immune-regulating, and cardiovascular disease-alleviating effects (23). Chinese herbal medicines contain a variety of active ingredients, including polysaccharides, alkaloids, amino acids, vitamins, and more (24). These components can enhance animal performance by boosting the body’s anti-apoptotic (25), anti-inflammatory, and antioxidant capabilities (26), as well as by modulating microbial composition (27). Previous research has shown that adding herbal mixture (*Paeonia lactiflora, licorice, dandelion*, and *tea polyphenols*) can enhance growth performance, boost immunity, and alter the composition of the intestinal microbiota in weaning pigs (28). Certain studies conducted on pigeons have discovered that the addition of AEF (*Astragalus, Epimedium*, and *Ligustrum lucidum*) extract to their drinking water resulted in improved intestinal health and enhanced growth performance, especially under conditions of stress pigeons (29). A herbal mixture made up of numerous herbs includes multiple active components that may be more effective biologically than a single herb, as well as having a wider range of applications and better production results (28, 42).

In the animal body, the intestinal microbiota not only governs the digestion and absorption of nutrients (30) but also influences immune homeostasis and chronic diseases (31). It’s worth noting that the gut microbiota can be passed from the mother to her offspring through vertical transmission (32). The transmission of the microbiota is influenced by the microbiome composition of maternal hens’ feces, embryos, and chicks’ ceca. Maternal nutritional strategies play a critical role in regulating the phenotypic traits of animal offspring, and ensuring proper maternal nutrition is essential for early embryonic development (33). Furthermore, the nutrients in eggs are necessary for the early growth and development of chicks. There is a hypothesis that alterations in the maternal diet might affect the nutrient constituents of the eggs, consequently influencing the nutritional requirements and gut microbiota of chicken offspring (34).

To date, the combination of CHMs (*Epimedium, Angelica sinensis, Rehmanniae Radix Preparata, Radix Bupleuri, Pericarpium citri reticulatae, Leonurus japonicus, Radix Paeoniae Alba, Eucommia ulmoides, Lycii fructus, Codonopsis pilosula*, and *Astragalus membranaceus*) has not been extensively studied in Wenchang Breeder Hens. Furthermore, the potential influence of maternal supplementation with CHMs on the meconium microbiota of offspring has yet to be fully understood. The objective of this study was to explore the potential benefits of maternal supplementation with CHMs on various aspects, including productive performance, egg quality, immune status, anti-apoptosis ability, and intestinal microbiota, and to investigate the potential influence on the meconial microbiota structure in offspring chicks.

## 2 Materials and methods

### 2.1 Preparation of Chinese herbal mixtures

The raw materials of Chinese herbal mixtures were purchased from Hebei Anguo Qi’an Pharmaceutical Co. Ltd. in China, and then the herbs were crushed into fine powder, sifted through an 80-mesh sieve, and mixed thoroughly in proportion and stored at room temperature (25°C) for later use. The CHMs has the following contents per 100 g: *Epimedium* 10.5 g, *Angelica sinensis* 10.5 g, *Rehmanniae Radix Preparata* 5.3 g, *Radix Bupleuri* 5.3 g, *Pericarpium citri reticulatae* 5.3 g, *Leonurus japonicus* 15.8 g, *Radix Paeoniae Alba* 5.3 g, *Eucommia ulmoides* 10.5 g, *Lycii fructus* 10.5 g, *Codonopsis pilosula* 10.5 g, and *Astragalus membranaceus* 10.5 g in the ratio 2:2:1:1:1:1:3:1:2:2:2:2:2. The proximate chemical composition of CHMs was determined using AOAC (Association of Official Analytical Chemists) procedures (35), which included crude protein, ether extract, crude fiber, ash, total phosphorus, and calcium. The total polysaccharides in CHMs were determined using the phenol-sulfuric acid method with glucose as the standard (36). The active ingredients and nutrient composition of CHMs are shown in Table 1.

**Table 1:** The nutritive composition and active ingredients of CHMs powder.

Nutritive ingredients	Content (%)	Effective active ingredients	Content (mg/g)
Crude protein	8.93	Total polysaccharides (%)	12.65
Crude fat	2.7		
Crude fiber	15.4		
Ash	6.5		
Ca	0.64		
P	0.21		

### 2.2 Animals, diet, and experimental design

All experimental protocols were approved by the Animal Care and Use Committee of the South China Agricultural University (approval number: SYXK 2019-0136, Guangzhou, China).

A total of 168 thirty-week-old Wenchang breeder hens were randomly assigned to two groups, each consisting of six replicates, with fourteen hens in each replicate. These Wenchang breeder hens were sourced from Enping Jilong Industrial Co., Ltd. (Jiangmen, China) and were housed and fed at the Enping Jilong hens production facility. The groups were fed a basal diet (CON group) and a basal diet with 1,000 mg/kg CHMs (CHMs group) for 10 weeks. The photoperiod was maintained at 16 h of light and 8 h of darkness throughout the study. Each hen received 88 g of feed per day to prevent overfeeding and had unlimited access to fresh water. Breeder hens underwent artificial insemination, with 35 μL of pooled semen administered to each bird every 3 days, following the procedure outlined by Liu et al. (37). The ingredient and nutrient composition of the basic diets was shown in Table 2.

**Table 2:** The ingredient and nutrient composition of the basal diet (% as fed basis).

Ingredients	Content (%)
Corn	57.80
Soybean meal	23.90
Fish meal	3.40
Soybean oil	1.60
Limestone	6.85
Gypsum Powder	0.65
Calcium hydrogen phosphate	1.20
Uniform chaff	3.60
Premixes^a^	1.00
Total	100
Nutrient composition^b^	
Digestible energy (MJ/kg)	11.31
Crude protein (%)	17.03
Calcium (%)	3.14
Available phosphorus (%)	0.36
SID-Lys (%)	0.94
SID-Met (%)	0.41
SID-Cys (%)	0.26

a The premix per kilogram of feed contains vitamin A 16500 IU, vitamin D 36250 IU, vitamin E 75 IU, vitamin K3 10 mg, vitamin B1 5 mg, vitamin B2 15 mg, vitamin B6 15 mg, vitamin B12 0.05 mg, vitamin C 186 mg, folic acid 2.5 mg, D-biotin 0.375 ng, nicotinamide 100 mg, DL-tocopheryl acetate 40 mg, Fe 200 mg, Cu 16.66 mg, Mn 184 mg, Zn 150 mg, I 0.834 mg, Se 0.416 mg, choline chloride 0.75 g, DL-methionine 1.188 g, DL-lysine 0.591 g, NaHCO_3_ 1.485 g, NaCl 2.39 g, phytase 1,500 IU, xylanase 1,500 IU, cellulase 250 IU, acid protease 125 IU, Amylase 25,000 IU, β-mannanase 4,500 IU, β-glucanase 1,500 IU.

b The nutrient levels were calculated from data provided by Feed Database in China.

### 2.3 Sample collection

At the end of the feeding period, one hen per replicate (a total of six hens) was randomly selected and subjected to a 12 h fasting period for sample collection. Blood samples were drawn from the wing vein into tubes and allowed to stand at room temperature for 20 min. Subsequently, the tubes were centrifuged at 3000 × g for 10 min at 4°C to collect the serum. These serum samples were stored at −20°C for future analysis. Hens were euthanized by exsanguination and necropsied, and the liver, ovary, and mucosal samples of the middle jejunum were collected immediately and quickly frozen at −80°C for further analysis.

In the final week of the experiment, eggs from each group were collected and subsequently incubated using the same incubator. After hatching, meconium samples were collected from the offspring chicks by gently massaging their abdominal regions. Both cecal digesta and meconium samples were aseptically collected and then stored at −80°C for 16S rRNA analysis.

### 2.4 Measurement of the laying and hatching performance and egg quality

Laying performance was assessed throughout the experiment by daily recording of egg production, egg weight, and the number of qualified eggs. Laying rate, average egg weight, qualified egg rate, and feed conversion rate were calculated based on the data collected from each replicate.

At the conclusion of the experimental period, a random selection of 10 eggs from each replicate was used to evaluate egg quality. We measured both the horizontal and vertical diameters of these selected eggs using a Vernier caliper (530-101, Mitutoyo, Japan) to calculate the egg shape index. Eggshell strength was determined with an eggshell strength tester (ESG-1, Yaoen, Nanjing, China), while eggshell thickness and weight were measured separately using a Vernier caliper and an electronic balance (FB224, Hengping, Shanghai, China), respectively. Furthermore, egg yolk color, albumen height, and the Haugh unit were assessed using an automatic egg quality tester (EA-01, Orka, Israel).

For hatching performance assessment, all eggs were incubated in the same incubator (Bengbu Sanyuan Incubation Equipment Co., Ltd., Anhui, China) during the experimental period. The incubator maintained a temperature range of 37.2°C to 38.0°C and a relative humidity of 60 to 75%. Eggs were manually turned 12 times a day throughout the incubation period and were lightly sprayed with water once daily, starting from the 15th day of incubation until they hatched (38). The parameters, including fertility, hatching of fertile eggs, hatchability of set eggs, embryo mortality, and the weight of the hatched chicks, were recorded and calculated.

### 2.5 Measurements of fatty acid content of egg yolk

At the conclusion of the experimental period, five eggs per replicate were randomly selected to assess the fatty acid content in the egg yolk. The fatty acid composition of the samples was determined using gas chromatography. The mean level of each fatty acid was then used to calculate the total content of saturated fatty acids (SFA), monounsaturated fatty acids (MUFA), and polyunsaturated fatty acids (PUFA). Fatty acid measurement services were provided by Waltek Testing Group (Foshan) Co., Ltd.

### 2.6 Analysis of biochemical components in serum

The activities of caspase-8 (caspase-8; Detection range 83.75 pmol/L ~ 120 pmol/L), the serum concentration of immunoglobulin A (IgA; Detection range 10 μg/mL ~ 320 μg/mL), immunoglobulin G (IgG; Detection range 75 μg/mL ~ 2,400 μg/mL), and interleukin-6 (IL-6; Detection range 1 pg./mL ~ 32 pg./mL) were examined by Enzyme-linked immunosorbent assay (ELISA). All ELISA kits were purchased from Shanghai Enzyme-linked Biotechnology Co., Ltd. (China). Serum concentrations of IgA, IgG, IL-6, and Caspase 8 activity were measured following the instructions of the ELISA kit.

### 2.7 Real-time quantitative polymerase chain reaction (RT-qPCR)

Total RNA was extracted from tissue samples (liver, ovary, and jejunal mucosa) using the RNA isolator Total RNA Extraction Reagent kit (Vazyme). Subsequently, total cDNA was synthesized using HiScript III RT SuperMix for qPCR (+gDNA wiper) (Vazyme) with 1 μg of total RNA, followed by RT-qPCR amplification using the ChamQ universal SYBR qPCR Master Mix (Vazyme). Gene primers for qRT-PCR were designed by Primer Premier 6.0 software (Premier Biosoft International, United States), synthesized by Tsingke Biotechnology Co., Ltd. (Beijing, China), and used in this study (Table 3). The relative expression of the target gene was analyzed using the 2^−ΔΔCt^ method, normalized against the geometric mean of the expression of β-actin and GAPDH (39).

**Table 3:** Laying performance of breeder hens.

Gene	Primer sequence (5′ → 3′)	Accession number
β-actin	Forward: GAGAAATTGTGCGTGACATCA	L08165.1
	Reverse: CCTGAACCTCTCATTGCCA	
Bax	Forward: GGTGACAGGGATCGTCACAG	XM_422067
	Reverse: TAGGCCAGGAACAGGGTGAAG	
Bcl-2	Forward: GCTGCTTTACTCTTGGGGGT Reverse: CTTCAGCACTATCTCGCGGT	NM_205339.2
Caspase8	Forward: TAAAATGACCAGCCGACCCC	NM_204592.4
	Forward: TCTGCATCCACATGTGTCCC	
GAPDH	Reverse: ACCTCTGTCATCTCTCCACA	K01458
	Reverse: GGCAGAGCTCAGTGTCCATT	
IL-1β	Forward: CAGCCTCAGCGAAGAGACCTT Reverse: ACTGTGGTGTGCTCAGAATCC	NM_204524.2
IL-4	Forward: CTTCCTCAACATGCGTCAGC	AJ621735
	Reverse: TGAAGTAGTGTTGCCTGCTGC	
MyD88	Forward: ATCCGGACACTAGAGGGAGG	NM_001030962.1
	Reverse: GGCAGAGCTCAGTGTCCATT	
NF-κB	Forward: GTGTGAAGAAACGGGAACTG	NM_205129
	Reverse: GGCACGGTTGTCATAGATGG	
TLR4	Forward: AGGCACCTGAGCTTTTCCTC	NM_001030693.1
	Reverse: TACCAACGTGAGGTTGAGCC	

### 2.8 Cecal and meconial microbiota analysis in breeder hens and offspring chicks

This trial followed the established procedures of previous researchers (40). To isolate total microbial DNA from the cecal and meconial content samples, we utilized the QIAamp DNA Stool Kit (Qiagen, Valencia, United States). The V3–V4 region of the bacterial 16S rRNA gene was amplified through PCR with the following primers: forward primer 5′-ACTCCTACGGGAGGCAGCA-3′ and reverse primer 5′-GGACTACHVGGGTWTCTAAT-3′. PCR products were then purified using Vazyme VAHTS^™^ DNA Clean Beads (Vazyme) and quantified with a PicoGreen dsDNA Assay Kit (Invitrogen, United States). Subsequently, we conducted 16S rRNA sequencing on the Illumina Novaseq_PE250 platform (Illumina), with sequencing services provided by Personal Biotechnology Co., Ltd. in Shanghai, China. Data collection and analysis were carried out using the Genescloud Platform^1^.

### 2.9 Statistical analysis

All data were initially organized using Excel software and subsequently subjected to statistical analysis with SPSS 20 and GraphPad Prism 7.0 software. For the comparison of data between two groups, a two-tailed unpaired Student’s *t*-test was employed. The data are presented as the means ± SEM. Significance was determined at *p* < 0.05.

## 3 Results

### 3.1 Effects of dietary CHMs on laying performance of breeder hens

Regarding the laying performance of breeder hens, as presented in Table 4, there was no significant difference was observed in the percentage of qualified eggs between the CON and CHMs groups. However, the CHMs group showed a significant increase in laying rate and average egg weight (*p* < 0.05) compared to the CON group, along with a decrease in the feed conversion ratio (*p* < 0.05).

**Table 4:** Laying performance of breeder hens.

Items	CON	CHMs	SEM	Value of *p*
Laying rate, %	61.92	65.62*	0.081	0.014
Average egg weight, g	43.09	44.37*	0.311	0.032
Percentage of qualified eggs, %	94.82	95.70	0.030	0.155
Feed conversion ratio, g/g	3.32	3.08*	0.055	0.015

*Significant differences in comparison with the CON group are expressed as *p < 0.05 and **p < 0.01 (*n* = 6).

### 3.2 Effects of dietary CHMs on hatchability of breeder hens

The fertilizing capacity and hatchability results were presented in Table 5. The embryo mortality of the CHMs group was significantly decreased (*p* < 0.05) than that of the CON group. In addition, supplementing CHMs to the diet led to a significant increase in the hatching of fertile eggs and the weight of offspring chicks compared to the CON group (*p* < 0.05).

**Table 5:** Fertilizing capacity and hatchability of breeder hens.

Items	CON	CHMs	SEM	Value of *p*
Embryo mortality, %	7.61	3.16*	1.173	0.047
Fertility of set eggs, %	93.00	94.00	1.067	0.667
Hatch of fertile eggs, %	92.39	96.84*	1.173	0.045
Hatchability of set eggs, %	86.00	90.00	1.675	0.143
Offspring chick’s weight, g	31.93	32.90*	0.230	0.023

*Significant differences in comparison with the CON group are expressed as *p < 0.05 (*n* = 6).

### 3.3 Effects of dietary CHMs on egg quality of breeder hens

Concerning the egg quality indices shown in Table 6, the CHMs in diet did not affect egg shape index, eggshell thickness, eggshell ratio, egg yolk colour and yolk percentage at 10 weeks. However, CHMs significantly improved eggshell strength (*p* < 0.05), albumen height (*p* < 0.01) and haugh unit (*p* < 0.05).

**Table 6:** Egg quality of breeder hens.

Items	CON	CHMs	SEM	Value of *p*
Egg shape index	1.33	1.31	0.010	0.534
Eggshell thickness, mm	0.32	0.32	0.004	0.937
Eggshell strength, kg/cm^2^	3.76	4.30*	0.121	0.023
Eggshell ratio, %	13.11	13.20	0.248	0.850
Albumen height, mm	2.51	3.21**	0.139	0.009
Yolk colour	5.38	5.88	0.205	0.227
Haugh unit	48.07	56.42*	1.732	0.013
Yolk percentage, %	32.47	32.88	0.713	0.695

*Significant differences in comparison with the CON group are expressed as *p < 0.05 and **p < 0.01 (*n* = 12).

### 3.4 Effects of dietary CHMs on fatty acid content in egg yolk

The fatty acid contents of egg yolk from breeder hens fed with CHMs were presented in Table 7. Regarding SFA, compared to the CON group, CHMs significantly increased margaric acid (C17:0) in the yolk (*p* < 0.05), and we also observed a tendency to increase behenic acid (C22:0, *p* = 0.086). Furthermore, there was a significant increase in cis-11-Eicosenoic acid (C20:1), a type of monounsaturated fatty acid (MUFA), in the CHMs group (*p* < 0.05). In the case of polyunsaturated fatty acids (PUFAs), CHMs-supplemented diets resulted in a significant increase in the content of dihomo γ-linolenic acid (C20:3n-6) and docosatetraenoic acid (C20:4n-6) in egg yolk (*p* < 0.05).

**Table 7:** Fatty acid content in egg yolk of breeder hens.

Items	CON	CHMs	SEM	*p*-value
C14:0	0.040	0.045	0.002	0.341
C15:0	0.004	0.005	0.001	0.253
C16:0	1.830	2.118	0.157	0.390
C17:0	0.013	0.023*	0.003	0.046
C18:0	1.338	1.574	0.137	0.421
C22:0	0.013	0.019	0.002	0.086
Total SFA	3.238	3.784	0.293	0.382
C14:1	0.007	0.007	0.0006	0.919
C16:1	0.317	0.332	0.026	0.815
C17:1	0.006	0.008	0.001	0.450
C18:1n-9	5.698	5.414	0.540	0.810
C20:1	0.049	0.065*	0.004	0.019
C24:1	0.035	0.036	0.006	0.925
Total MUFA	6.112	5.862	0.567	0.840
C20:2	0.016	0.018	0.001	0.616
C18:3n-3	0.022	0.024	0.002	0.635
C20:3n-3	0.234	0.208	0.043	0.782
C22:6n-3	0.155	0.221	0.024	0.186
C18:2n-6	1.964	2.328	0.171	0.314
C18:3n-6	0.017	0.021	0.002	0.317
C20:3n-6	0.006	0.012*	0.001	0.022
C20:4n-6	0.067	0.156*	0.022	0.028
Total PUFA	2.481	2.987	0.219	0.273

*SFA, saturated fatty acid; MUFA, monounsaturated fatty acid; PUFA, polyunsaturated fatty acid. Significant differences in comparison with the CON group are expressed as *p < 0.05 (*n* = 5).

### 3.5 Effects of dietary CHMs on immunoglobulins and inflammatory factors of breeder hens

**Figure 1:**
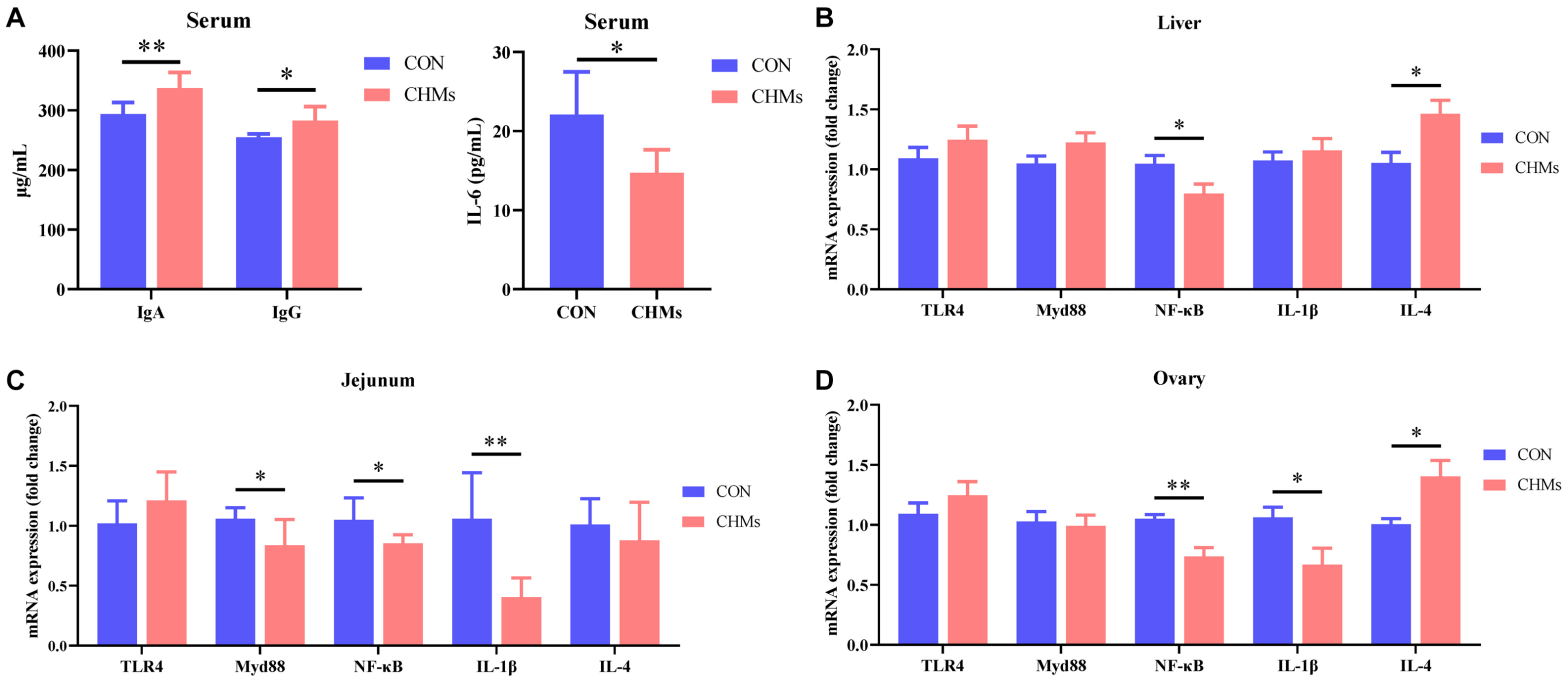
Effects of dietary CHMs on immunoglobulins and inflammatory factors of breeder hens. **(A)** Serum IgA, IgG; **(B)** Serum IL-6; **(C,D)** The expressions of inflammatory genes (TLR4, MyD88, NF-κB, IL-1β, IL-4) in liver, jejunum and ovary, respectively. The data were expressed as mean ± SEM (*n* = 6). *p < 0.05 and **p < 0.01 vs. CON group.

To further investigate the impact of CHMs on immune function, we assessed the levels of immunoglobulins and inflammatory factors in breeder hens (Figure 1). The serum concentrations of IgA and IgG in the CHMs group were significantly higher than those in the CON group (*p* < 0.01 and *p* < 0.05, respectively). With the introduction of CHMs, the serum levels of the proinflammatory cytokine IL-6 in breeder hens were significantly lower than those in the CON group (*p* < 0.05). Moreover, dietary supplementation with CHMs led to increased mRNA expression of IL-4 (*p* < 0.05 and *p* < 0.05, respectively) and reduced mRNA expression of NF-κB (*p* < 0.05 and *p* < 0.01, respectively) in both the liver and ovary. It also resulted in decreased mRNA expression of IL-1β in the ovary of breeder hens compared to the CON group (*p* < 0.05). In the jejunal mucosa of breeder hens, CHMs feed supplementation significantly decreased the relative mRNA levels of MyD88, NF-κB, and IL-1β (*p* < 0.05, *p* < 0.05, and *p* < 0.01, respectively) compared to the CON group.

### 3.6 Effects of dietary CHMs on apoptosis-related factors of breeder hens

**Figure 2:**
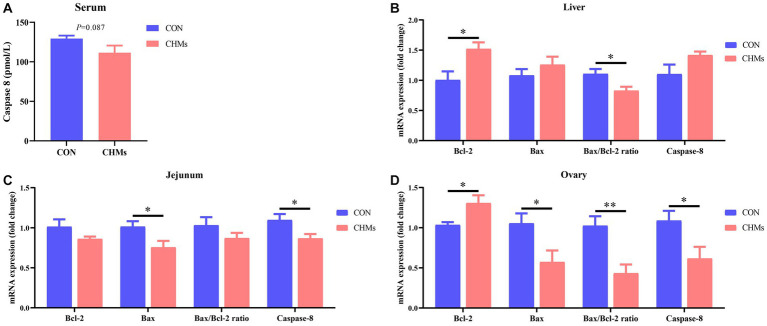
The impact of dietary CHMs on apoptosis-related factors in breeder hens. **(A)** Serum Caspase-8 concentration; **(B–D)** Relative mRNA expression of apoptosis-related genes in the live, jejunum and ovary, including Bcl-2, Bax, Bax/Bcl-2 ratio and Caspase-8. The data were expressed as mean ± SEM (*n* = 6). *p < 0.05 and **p < 0.01 vs. CON group.

As shown in Figure 2, in the serum of breeder hens fed with dietary CHMs, there was a trend towards lower Caspase-8 concentrations (*p* = 0.087). Additionally, a significant decrease in the mRNA expression of Caspase-8 was observed in the jejunal mucosa and ovary of the CHMs group (*p* < 0.05). In comparison to the CON group, the mRNA expression of the anti-apoptotic gene Bcl-2 showed a significant increase (*p* < 0.05), and the Bax/Bcl-2 ratio was significantly down-regulated (*p* < 0.05) in the liver of breeder hens in the CHMs group. Additionally, in the jejunal mucosa, the CHMs group exhibited a higher relative mRNA expression of the pro-apoptotic gene Bax compared to the CON group (*p* < 0.05). In the ovary of breeder hens, the CHMs group had higher expression of Bcl-2 (*p* < 0.05) and lower expression of Bax (*p* < 0.05) compared to the CON group, resulting in a highly significant downregulation of the Bax/Bcl-2 ratio (*p* < 0.01).

### 3.7 Description of the 16S rRNA gene sequencing data

**Figure 3:**
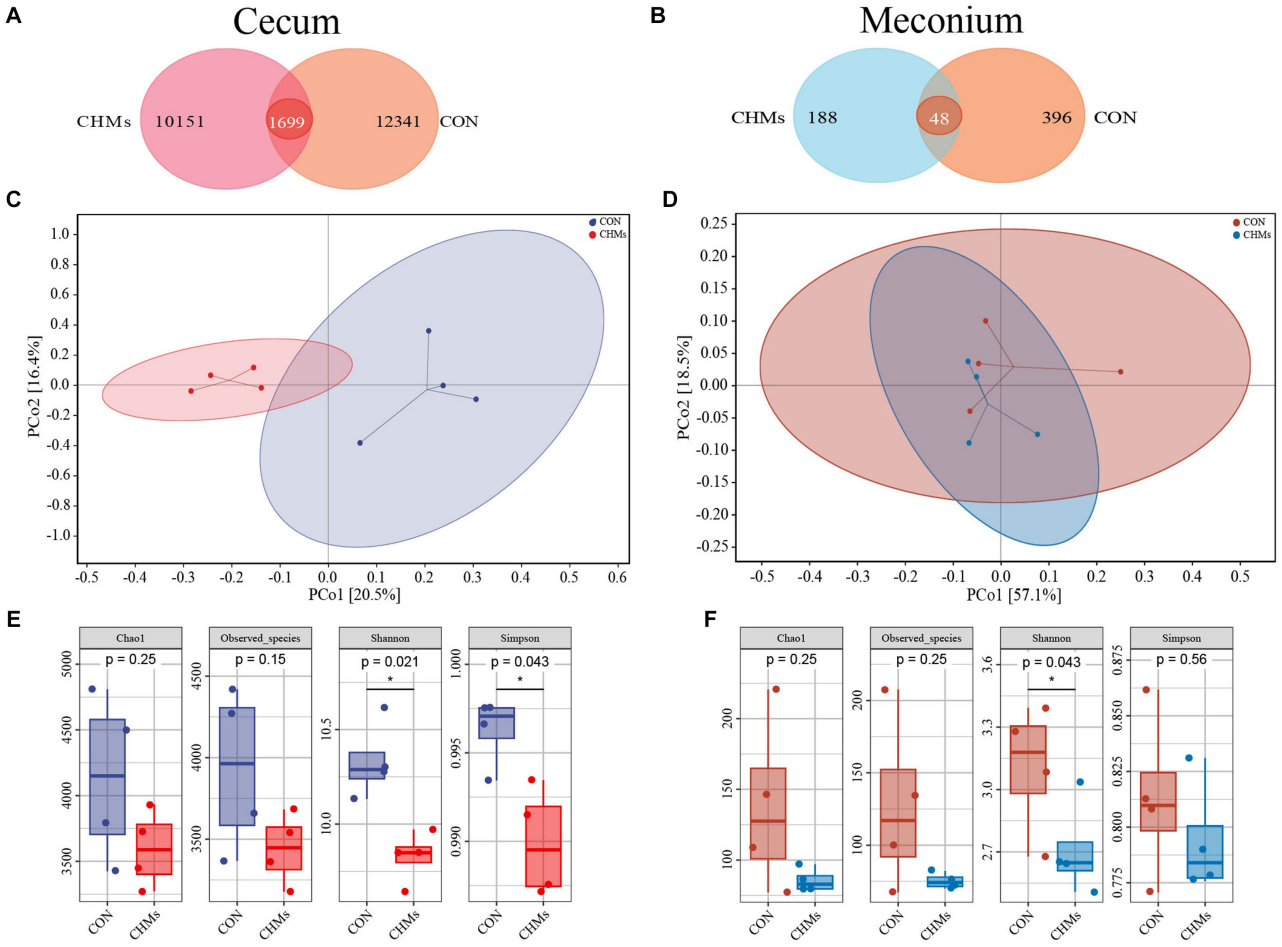
Gut microbiota diversity of breeder hens and offspring chicks. **(A,B)** Venn diagram of caecal and meconial microbiota on OTUs level. **(C,D)** PCoA two-dimensional figure based on Bray_Curtis distance analysis. **(E,F)** Alpha diversity, including Chao 1, Observed_species, Shannon index and Simpson index. The data were expressed as mean ± SEM (*n* = 4). *p < 0.05, vs. CON group.

To explore the effects of dietary supplementation with CHMs on the gut microbiota, we performed 16S rRNA gene sequencing on the cecal content and meconium of breeder hens and offspring chicks, respectively. The diversity of the cecal and meconial microbiota was shown in Figure 3. Venn diagram analysis revealed that 1,699 OTUs were overlapped among the two groups, and 12,341, and 10,151 specific OTUs were unique to the CON and CHMs groups on the cecal microbiota, respectively (Figure 3A). Concerning the meconial microbiota of offspring chicks, a Venn diagram analysis revealed 48 shared OTUs between the CON and CHMs groups. The CON group exhibited 396 unique OTUs, while the CHMs group had 188 unique OTUs (Figure 3B). Based on the PCA (principal component analysis) scatterplot, a clear separation was observed between the cecal samples of the CON and CHMs groups, with no overlapping clusters (Figure 3C). However, there was no obvious separation between the meconium samples of CON and CHMs groups (Figure 3D). Then, we employed Chao 1, Observed_species, Shannon index, and Simpson index to assess the alpha diversity of the cecal and meconial microbiota. The Shannon and Simpson diagrams indicated that the *α* diversity of the cecal microbiota in breeder hens was visibly lower in the CHMs group than that in the CON group (*p* < 0.05) (Figure 3E). Furthermore, in comparison to the CON group, the CHMs group exhibited a decrease in the Shannon indices in the meconial microbiota (*p* < 0.05) (Figure 3F). No significant differences were observed in the Chao 1 and Observed_species indices of the cecal and meconial microbiota between the two groups (*p* > 0.05).

### 3.8 Changes in cecal and meconial microbiota communities

**Figure 4:**
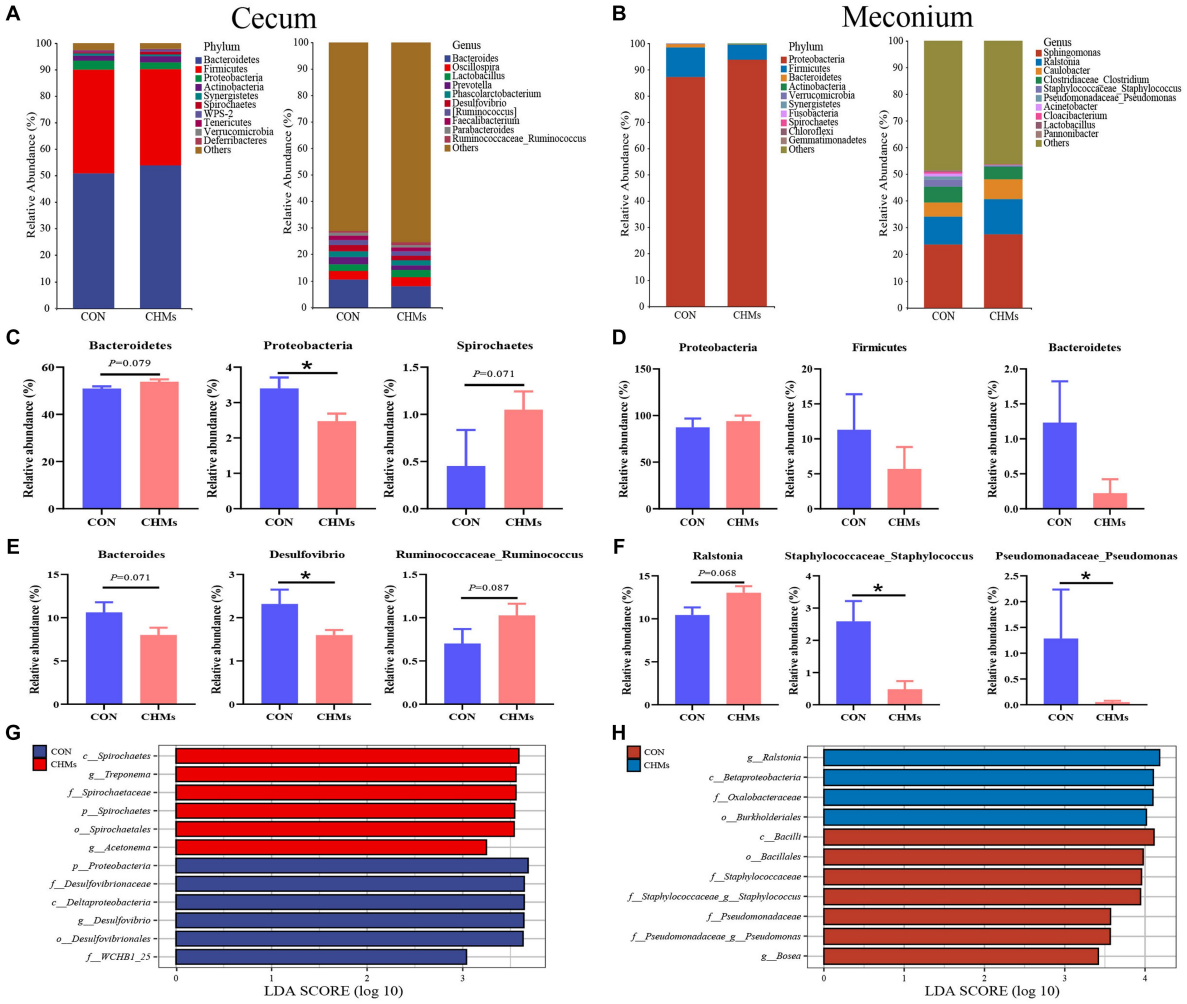
Gut microbiota structure of breeder hens and offspring chicks. **(A,B)** Relative abundance of caecal and meconial microbiota on phylum and genus level. **(C–F)** The significant difference genera and phylum between two groups on caecal and meconial microbiota, respectively. **(G,H)** LEfSE taxonomic cladogram between two groups on caecal and meconial microbiota (LDA > 3). The data were expressed as mean ± SEM (*n* = 4). *p < 0.05, vs. CON group.

To explore how maternal nutrition interventions, immunity, and microbes may play roles in maternal effects, we analyzed the changes in the composition of the cecal and meconial microbiota communities at the phylum and genus levels (Figure 4). In the cecal microbiota of breeder hens, the three most abundant bacterial phyla were *Bacteroidetes, Firmicutes*, and *Proteobacteria* (Figure 4A). The dominant genera included *Bacteroides*, *Oscillospira*, and *Lactobacillus*. At the phylum level, analysis revealed significantly lower abundances of *Proteobacteria* in the cecal microbiota of breeder hens in the CHMs group compared to the CON group (*p* < 0.05) (Figure 4C). However, the abundances of *Bacteroidetes* and *Spirochaetes* exhibited an upward trend in the CHMs group, respectively (*p* = 0.079, *p* = 0.071). Notably, at the genus level of the cecal microbiota, the intervention of CHMs significantly reduced the relative abundance of *Desulfovibrio* (*p* < 0.05) (Figure 4E). Compared to the CON groups, there was a decreasing trend in the relative abundance of *Bacteroides* in the CHMs group (*p* = 0.071), while the relative abundance of *Ruminococcaceae_Ruminococcus* showed an upward trend in the CHMs group (*p* = 0.087).

Next, we investigated the composition and structure of the meconial microbiota based on some maternal effects benefiting offspring fitness. In the meconial microbiota of offspring chicks, *Firmicutes* and *Bacteroidetes* emerged as the two most abundant bacterial phyla in both the CON and CHMs groups, accounting for over 98% of the total phyla (Figure 4B). At the genus level, *Sphingomonas* and *Ralstonia* were the dominant genera in the meconial microbiota, followed by *Caulobacter* and *Clostridiaceae_Clostridium*. No significant differences were observed in the bacterial phyla between the two groups (*p* > 0.05) (Figure 4D). In comparison to the CON group, the CHMs group exhibited a lower relative abundance of *Staphylococcaceae_Staphylococcus* and *Pseudomonadaceae_Pseudomonas* (*p* < 0.05) (Figure 4F). The relative abundance of Ralstonia showed an upward trend in the CHMs group (*p* = 0.068).

The Linear Discriminant Analysis (LDA) Effect Size (LEfSe) was employed to identify the major microflora with significant differences between the CON group and CHMs group. The LEfSe taxonomic cladogram of cecal microbes revealed that *Spirochaetes* (class), *Treponema* (genus), and *Spirochaetaceae* (family) exhibited higher abundance in the CHMs group (Figure 4G). While the *Proteobacteria* (phylum), *Desulfovibrionaceae* (family), and *Deltaproteobacteria* (class) were the most significant abundance of the CON group. Regarding the LEfSe analysis of meconial microbes, some bacterial groups, including the *Ralstonia* (family), *Betaproteobacteria* (class) and *Oxalobacteraceae* (family) in CHMs group had a higher score, whereas some other bacterial groups, such as *Bacilli* (class), *Bacillales* (order), *Staphylococcaceae* (family) in CON group had a higher score (Figure 4H).

### 3.9 Correlation analysis of altered intestinal microbe and different indicators

**Figure 5:**
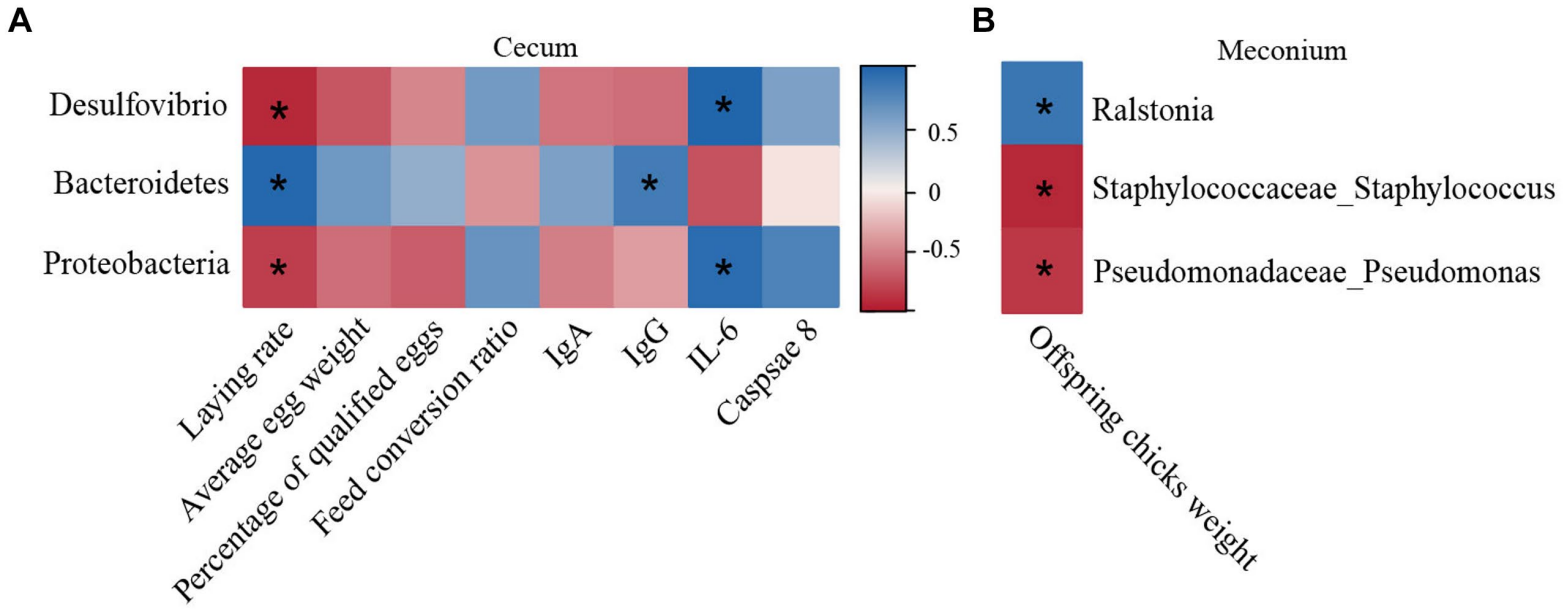
Matrix diagram of the correlation analysis. **(A)** Spearman correlation analysis among the cecal microbiota, laying performance, and serum indices. **(B)** Spearman correlation analysis between the meconial microbiota and weight of offspring chicks. Positive and negative correlations are shown by the blue and red matrices, respectively. *p < 0.05 (*n* = 4).

Potential correlations between the meconial and cecal microbiota alterations with many different indicators were evaluated employing a Spearman correlation analysis (Figure 5). The genera *Desulfovibrio* and the phylum *Proteobacteria* were negatively correlated with laying performance (*r* = −0.88, −0.83, *p* < 0.05) but positively correlated with the level of serum IL-6 (*r* = 0.95, 0.81, *p* < 0.05) (Figure 5A). Additionally, the level of serum IgG and laying performance were positively correlated with the phyla Bacteroidetes (*r* = 0.76, 0.90, *p* < 0.05). As shown in Figure 5B, the weight of offspring chicks had a connection positively with the abundances of *Ralstonia* (*r* = 0.76; *p* < 0.05), but it was negative correlated with *Staphylococcaceae_Staphylococcus* and *Pseudomonadaceae_Pseudomonas* (*r* = −0.81, −0.74, *p* < 0.05).

## 4 Discussion

In the current experiment with Wenchang Breeder Hens, we found that dietary supplementation with CHMs beneficially affected laying rate and average egg weight, although it reduced feed conversion rate. Despite the different combinations of Chinese herbal mixture (*Pine needle* and *Artemisia annua*), Li et al.’s study also reported that CHM increased egg production rates in hens but decreased feed conversion rates, which may be related to the regulation of cholesterol metabolism by CHM (41). Epimedium in CHMs has been shown to have multiple regulatory functions, including sexual dysfunction, hormone metabolism, immune function, and antioxidant properties (6). In our study, these great potentials were confirmed in the growth performance of laying hens. CHMs reduced embryonic mortality while increasing the hatching of fertile eggs and offspring chicks’ weight in this study. Therefore, the increase in egg production and improvement in hatching performance may be attributed to the improved health status of laying hens fed a diet supplemented with CHMs. However, due to the complexity of CHMs and the limitations of this study, further research is still needed to investigate their impact on animals.

Extracts of plant-derived polyphenols and polysaccharides demonstrate strong antioxidant activity, which effectively prevent protein breakdown by lowering oxidation of proteins and lipids (42). Early studies have suggested that the addition of Chinese herbs, specifically a combination of *R. astragali*, *S. miltiorrhiza Bunge*, and *C. monnieri*, led to a significant improvement of the albumen height and haugh unit in eggs (43). In terms of egg quality, dietary supplementation with CHMs significantly increased eggshell strength, albumin height, and haugh unit in this study. The CHMs contain a combination of polysaccharides and polyphenols derived from various Chinese herbs. It is plausible that the cumulative effects of these compounds contributed to the observed improvement in egg quality in this study. In addition, a significant increase was also observed in the content of margaric acid (SFA), cis-11-Eicosenoicacid (MUFA), dihomo γ-linolenic acid (n-6 PUFA), and docosatetraenoic acid (n-6 PUFA) in egg yolk. Previous studies have shown that the lipids in eggs, such as egg yolk oil, phospholipids, and fatty acids, are high-quality nutrients with anti-inflammatory, antioxidant, cardiovascular protection, memory improvement, and physiological homeostasis functions (44). Particularly, polyunsaturated fatty acids (PUFAs) are regarded as one of the most crucial components that influence the normal development and physiological functions of the body (45). Numerous studies have shown that C20:3n-6, a long-chain polyunsaturated fat, is considered one of the most important components of breast milk (46). The high level of C20:3n-6 makes eggs from hens treated with CHMs a better choice for pregnant women and infants. In general, the intervention of CHMs improved the external and internal quality of eggs, which is a benefit for subsequent production or marketing.

Numerous clinical studies have found that CHMs have pleiotropic mechanisms of action in reproductive tract diseases, regulating the expression of estrogen receptors, reactive oxygen species, inflammatory factors, and apoptosis-related proteins (47). Inflammatory damage and cell apoptosis caused by reproductive tract infections are common causes of reproductive dysfunction in laying hens (48, 49). The reproductive function of hens is closely related to the levels of inflammatory factors and apoptosis-related genes in the body, which is particularly important for layer production. Li et al.’s study found that Shaoyao decoction improves tissue fibrosis and treats radiation enteritis by reducing inflammation and apoptotic responses (50). The findings of this study suggest that CHMs tend to decrease the activity of caspase-8 enzyme in hens’ serum. The relative expression of genes for apoptosis-related proteins in liver, jejunum, and ovarian tissues was also determined in our study. CHMs regulated the expression of mRNAs including Bax, Bcl-2, Caspase-8 in the above three tissues and activated the anti-apoptotic responses in hens. In a study conducted by Yan et al., lotus leaf extract was observed to upregulate the mRNA expression levels of the Caspase-8, Caspase-9, and Caspase-10 in laying hens. Simultaneously, it downregulated the mRNA expression levels of Caspase-3 and Caspase-7. This regulation of caspases had an impact on cell apoptosis and immune function, ultimately resulting in an improvement in salpingitis among laying hens (51). CHMs diet could regulate the ability of ovarian cell apoptosis in breeder hens.

In recent years, the immunomodulatory, anti-inflammatory, and anti-apoptotic effects of Chinese medicines have been widely studied. In our study, CHMs treatment increased the concentrations of serum immunoglobulins IgA and IgG in hens, which are considered to potent inducers for promoting neutrophils against pathogens (52). In addition, CHMs decreased the concentration of the pro-inflammatory factor IL-6 in hens’ serum. The relative expression of genes for inflammation-related factors in liver, jejunum, and ovarian tissues was also determined in our study. CHMs regulated the expression of mRNAs including NF-κB, IL-4, IL-1β, and MyD88 in the above three tissues and activated the anti-inflammatory responses in hens. IgA, IgM, and IgG are the major serum immunoglobulins of the body as well as their concentrations in plasma have a significant relationship with immunity in laying hens (53). The interleukin (IL) family plays a crucial role in regulating inflammation, with IL-1β and IL-6 assuming vital roles in maintaining intestinal structural integrity, modulating the intestinal immune systems, and being involved with various apoptotic and inflammatory responses (54). Chinese herbal medicines (CHMs) contain numerous bioactive substances, including polysaccharides, volatile oils, flavonoids, alkaloids, and glycosides, which can enhance the body’s immunity (28). Therefore, we hypothesize that CHMs possess potent anti-inflammatory properties and the ability to regulate apoptosis protein-related genes, ultimately bolstering the immunity and health status of breeder hens.

It is widely known that the cecum of chickens contains a rich gut microbiota. Several studies have shown that herbal supplements or extracts have a beneficial effect on the composition of the intestinal microbiota in chickens. Evidently, the modulation of gut microbial structure by CHMs is intricately linked to the enhancement of growth performance, reproductive performance, and immune function in the organism (55, 56). The study by Song et al. demonstrated that dietary supplementation with astragalus polysaccharides significantly decreased the population of *Clostridium perfringens* in the cecum of broilers with necrotizing enteritis (57). In our study, CHMs altered the structure of the cecum microbiota in hens. Despite a decrease in the alpha diversity of the microbiota, there was an increase in beneficial bacteria (like *Ruminococcus*) and a decrease in pathogenic bacteria (like *Desulfovibrio*) in the cecum of hens. In the cecum of hens treated with CHMs, there was a significant decrease in *Desulfovibrio* (58), which is considered an intestinal noxious bacterium, while *Ruminococcus* (59), known for its capacity to degrade complex polysaccharides and transform them into a range of nutrients for their hosts, exhibited an inclination to increase. The fermented Chinese herbal compound containing Bupleurum chinense can reduce the abundance of *Desulfovibrio* in broiler cecum (27). Peng et al.’s (60) findings confirmed that supplementation of the diet with *Eucommia ulmoides* extract might simulate an immune response by altering gut microbial populations. On the one hand, this could be owing to the key bioactive ingredients of CHMs, such as polysaccharides, which have anti-inflammatory and immunomodulatory actions that enhance gut bacterial abundance. Another hand, and it could be the outcome of interactions between gut microbiota after CHMs intake.

Prior to egg laying, all the necessary components to support the growth of the offspring must be completely passed from the mother to the egg. We infer that CHMs produce favorable adaptive maternal effects to ensure the growth and intestinal health of the offspring by enhancing maternal immunity and anti-apoptotic effects as well as maintaining intestinal health, but their maternal effects in the offspring need to be investigated in depth. The immunity and health of the hen during laying influence the safety and development of the offspring embryo (61). Ding et al. (62) revealed that maternal microbial colonization occurs throughout embryonic development and claimed that the microbes present in the egg are transported from the oviduct during egg formation (despite the fact that they related the maternal fecal microbiota to the embryonic microbiota). Furthermore, consistent with the findings of the present study, previous research has demonstrated that meconium samples were predominantly composed of *Proteobacteria* and *Firmicutes*, with a lesser abundance of the phyla *Actinobacteriota* and *Bacteroidota* (63). *Staphylococcus* and *Pseudomonas* are both common foodborne pathogens and the most common causative agents of human septicemia (64, 65). The CHMs treatment led to a significant reduction in the relative abundance of *Staphylococcus* and *Pseudomonas* in the meconium of offspring. Gut microbes exert a profound impact on various physiological functions of the host, encompassing aspects from energy metabolism to immune responses, especially those related to the gut system of immunity (66). The gut microbes of newly hatched chicks were sourced from the maternal magnum, which was transferred into egg whites for colonization of the embryonic gut (61). Before long, environmental, genetic, and other factors shape the unique gut microbiota of offspring chicks. Current studies on *Sphingomonas* and *Ralstonia* have focused on botany and environmental soils since it can help plants to combat stressful environments (67, 68), but its role in the poultry gut microbiota remains to be investigated.

We performed Spearman correlation analysis of the significantly changed gut microbiota with phenotypic and physiological indicators. We observed a significant correlation between the hen’s egg production rate and the hen’s cecum microbiota, as well as body weight and meconium bacteria of offspring chicks. The reduction of harmful bacteria by the intervention of CHMs was negatively correlated with laying rate and chick body weight, which is consistent with the widely prevailing view (42). Therefore, we concluded that the addition of CHMs to diets maintains hen health by modulating the structure of the gut microbiota and reducing the relative abundance of opportunistic pathogens. Meanwhile, the resulting adaptive maternal effect maintains the initial weight and intestinal health of the offspring.

## 5 Conclusion

To summarize, this study showed that the addition of CHMs to laying hen diets can improve laying rate, hatchability, and eggs’ external and nutritional quality. We suggest that these positive effects are related to the expression of mRNAs for immunoglobulins, apoptosis-related proteins, and inflammatory factors in hens, along with changes in the gut microbiota. Notably, CHMs significantly reduced the relative abundance of opportunistic pathogens in the hens’ cecum and offspring’s meconium, which may account for the improved performance and egg quality, immunity, anti-apoptosis level, and anti-inflammatory level as well as further drive the maternal effects of Wenchang breeders by CHMs. In conclusion, these findings suggest that CHMs are a valuable feed additive for breeder hens.

## Data availability statement

The datasets presented in this study can be found in online repositories. The names of the repository/repositories and accession number can be found below: https://www.ncbi.nlm.nih.gov/sra/PRJNA1033218.

## Ethics statement

The animal studies were approved by Animal Care and Use Committee of the South China Agricultural University. The studies were conducted in accordance with the local legislation and institutional requirements. Written informed consent was obtained from the owners for the participation of their animals in this study.

## Author contributions

ML: Data curation, Formal analysis, Investigation, Methodology, Writing – original draft, Writing – review & editing. JH: Conceptualization, Visualization, Writing – original draft. MM: Investigation, Methodology, Writing – review & editing. GH: Investigation, Methodology, Software, Writing – review & editing. YZ: Data curation, Investigation, Writing – review & editing. YD: Investigation, Methodology, Software, Writing – review & editing. QQ: Project administration, Supervision, Writing – review & editing. WL: Conceptualization, Project administration, Supervision, Writing – review & editing. SG: Conceptualization, Funding acquisition, Writing – review & editing.

## Footnotes

^1^ www.genescloud.cn
